# Hydrogen Recovery by Mixed Matrix Membranes Made from 6FCl-APAF HPA with Different Contents of a Porous Polymer Network and Their Thermal Rearrangement

**DOI:** 10.3390/polym13244343

**Published:** 2021-12-11

**Authors:** Cenit Soto, Edwin S. Torres-Cuevas, Alfonso González-Ortega, Laura Palacio, Pedro Prádanos, Benny D. Freeman, Ángel E. Lozano, Antonio Hernandez

**Affiliations:** 1 Surfaces and Porous Materials (SMAP), Associated Research Unit to CSIC, Facultad de Ciencias, University of Valladolid, Paseo Belén 7, E-47011 Valladolid, Spain; marveliacenit.soto@uva.es (C.S.); laura.palacio@uva.es (L.P.); ppradanos@uva.es (P.P.); 2 Institute of Sustainable Processes (ISP), Dr. Mergelina s/n, E-47011 Valladolid, Spain; 3 McKetta Department of Chemical Engineering, Texas Materials Institute, The University of Texas at Austin, 200 E Dean Keeton St., Austin, TX 78712, USA; edwinstorres@utexas.edu (E.S.T.-C.); freeman@che.utexas.edu (B.D.F.); 4 Department of Organic Chemistry, School of Sciences, Facultad de Ciencias, University of Valladolid, Paseo Belén 7, E-47011 Valladolid, Spain; alfonso.gonzalez.ortega@uva.es; 5 Departament of Macromolecular Chemistry, Institute for Polymer Science and Technology (ICTP-CSIC), Juan de la Cierva 3, E-28006 Madrid, Spain; 6 IU CINQUIMA, University of Valladolid, Paseo Belén 5, E-47011 Valladolid, Spain

**Keywords:** mixed matrix membranes, gas separation, hydrogen separation, thermal rearrangement, porous polymer network

## Abstract

Mixed matrix membranes (MMMs) consisting of a blend of a hydroxypolyamide (HPA) matrix and variable loads of a porous polymer network (PPN) were thermally treated to induce the transformation of HPA to polybenzoxazole (β-TR-PBO). Here, the HPA matrix was a hydroxypolyamide having two hexafluoropropyilidene moieties, 6FCl-APAF, while the PPN was prepared by reacting triptycene (TRP) and trifluoroacetophenone (TFAP) in a superacid solution. The most probable size of the PPN particles was 75 nm with quite large distributions. The resulting membranes were analyzed by SEM and AFM. Up to 30% PPN loads, both SEM and AFM images confirmed quite planar surfaces, at low scale, with limited roughness. Membranes with high hydrogen permeability and good selectivity for the gas pairs H_2_/CH_4_ and H_2_/N_2_ were obtained. For H_2_/CO_2_, selectivity almost vanished after thermal rearrangement. In all cases, their hydrogen permeability increased with increasing loads of PPN until around 30% PPN with ulterior fairly abrupt decreasing of permeability for all gases studied. Thermal rearrangement of the MMMs resulted in higher permeabilities but lower selectivities. For all the membranes and gas pairs studied, the balance of permeability vs. selectivity surpassed the 1991 Robeson’s upper bound, and approached or even exceeded the 2008 line, for MMMs having 30% PPN loads. In all cases, the HPA-MMMs before thermal rearrangement provided good selectivity versus permeability compromise, similar to their thermally rearranged counterparts but in the zone of high selectivity. For H_2_/CH_4_, H_2_/N_2_, these nonthermally rearranged MMMs approach the 2008 Robeson’s upper bound while H_2_/CO_2_ gives selective transport favoring H_2_ on the 1991 Robeson’s bound. Thus, attending to the energy cost of thermal rearrangement, it could be avoided in some cases especially when high selectivity is the target rather than high permeability.

## 1. Introduction

The eventual depletion of the world’s fossil fuel reserves and growing public concern about climate change have been caused by soaring levels of carbon dioxide in the atmosphere. This is prompting calls for new, clean, and abundant energy sources. Many authors [1,2,3] have envisioned a hydrogen economy [4] based on energy from renewable sources and on hydrogen as a method of storing and transporting such primary energy [5]. Besides, hydrogen can be used as an input to H_2_ fuel cell systems, which operate with a relatively high efficiency without carbon oxides being produced [6,7]. The European Commission foresees that for effective progress towards a zero-greenhouse-gas economy by 2050, hydrogen should play an essential role [8].

Hydrogen can be produced via water electrolysis by low carbon or renewable energy. However, although electrolysis could be integrated in a number of different hybrid systems [8], this technology currently requires further development to reach industrial scale-up to reduce operational costs [5,9]. Other useful sources of hydrogen include the following:

- Steam–methane reforming (SMR) [9]. This starts by reacting methane with water steam at 750–800 °C to produce syngas, which is a mixture of hydrogen (H_2_) and carbon monoxide (CO). In the second step, known as a water–gas shift (WGS), CO reacts with water over a catalyst to form H2 and carbon dioxide (CO_2_). Subsequently, carbon capture and storage or usage (CCS/CCU) can be used to prevent CO_2_ emissions to the atmosphere.
- Methane cracking separates CH_4_ at high temperatures under an inert atmosphere to produce elemental carbon (which precipitates) and H_2_ with no CO_2_ emissions [10]. This procedure requires unreacted CH_4_ to be separated from H_2_ and recirculated into the reactor.
- H_2_ can also be produced from coal gasification combined with carbon capture for use or storage [9]. The purification process of synthesis gas, obtained from steam reforming of natural gas, is a key step and CO_2_ separation from H_2_ plays a crucial role [11].
- Biogas/biomethane reforming and biomass gasification and pyrolysis can also be a source of hydrogen from industrial or agriculture waste [12,13,14]. For instance, in dark fermentation, different proportions of H_2_, CO_2_, and CH_4_, depending on the microorganisms used, need to be purified.

Hydrogen saving is also relevant. For example, ammonia synthesis [15] involves the use of vast amounts of H_2_, because efficiency is low. The purge gas contains 60–70% H_2_ and 20–25% N_2_; therefore, recovering H_2_ from the purge gas and reusing it for ammonia synthesis or any other additional purpose is environmentally and economically beneficial [16] making the H_2_/N_2_ separation extremely interesting and important.

Different methods are accessible to separate H_2_ from complex gas mixtures. Among them, the most popular ones are pressure swing adsorption (PSA) and cryogenic methods. Cryogeny requires high-energy consumption [17] and pretreatment steps, to attain purity levels of nearly 99% [18]. PSA is currently responsible for up to 85% of the hydrogen produced worldwide [19] and can achieve 99.999% hydrogen purity, but only reaches 65–90% hydrogen recovery [18]. Both methods are complex and cannot be scaled up from low to high gas fluxes, thus, they lack flexibility [18,20].

Membrane-based separations are a proven alternative to conventional gas separation processes due to its high energy efficiency, low capital costs and footprint, along with its high robustness and its easy operation and maintenance. These advantages have been further enhanced with the new polymeric formulations proposed for hydrogen separations in the last 20 years [21]. In effect, at present, gas separation membranes have already been successfully used for H_2_/CO_2_ [19] and H_2_/CH_4_ [22] separations. Although this technology is already available at a commercial scale, further research in materials science is needed to prepare membranes with better and optimum balances between permeability and selectivity. In fact, currently polymeric membranes have a central presence in the membrane market for hydrogen separation, because they admit economical large-scale processing [23]. Among the many diverse types of polymeric membranes, as an evolution of blended and crosslinked polymers [24], MMMs are one of the most well-researched and promising approaches [25,26,27]. Chuah et al. [27] summarized the recent advances on MMMs with different emerging fillers that are potentially interesting for hydrogen separation. Mostly, researched fillers for MMMs include zeolites, graphene, or nanotubes; metal–organic frameworks (MOFs); and porous polymer networks (PPN) or covalent–organic frameworks (COFs).

Thermally rearranged (TR) polymeric matrices, using ortho-hydroxypolyimides (HPIs α-TR), ortho-hydroxypolyamides (HPAs β-TR), or other thermally rearrangeable polymers are promising materials thanks to their ability to develop hourglass-shaped cavities and to give unusually high selectivity [28]. Polymers of intrinsic microporosity (PIM) have also promising properties, although physical aging and plasticization apparently challenge them due to their high fractional free volume, which threatens their applicability for membrane gas separation [29]. TR polymers are specially resistant to plasticization but have low solubility in common solvents for membrane preparation, which is the main obstacle for their exploitation and industrialization, together with their high cost and the need for high-thermal-treatment temperature [30]. β-TR polymers are less expensive and need up to 100 °C less than α-TR to allow thermal rearrangement [31]. The lower rearrangement temperature of β-TR polymers is due to their hydroxyl groups in the initial HPA having higher mobility in comparison with the hydroxyl groups in HPIs required to obtain α-TR polymers. This is especially relevant attending to their scale-up due to the associated lower energy consumption. Moreover, β-TRs are suitable for separating small gases via size-sieving due to their micropore sizes being smaller than those of α-TRs [30,32].

Besides, the inclusion of nanoparticles with engineered pore volume, pore size, and/or surface adsorption properties [32] as nanoporous fillers to form Mixed Matrix Membranes (MMMs) can add fast and selective pathways for gas transport. In fact, it has been shown to improve membrane permeability, selectivity, or sometimes both. This improvement has been detected both for pure non-TR-able polymers [33] and also for thermally rearrangeable ones [34,35,36,37].

Recently, we blended some porous polymer networks (PPNs) [38] with both α and β-TR-able polymer matrices that resulted in high-performance gas separation materials with excellent balances of permeability and selectivity [35,38,39]. Our aim here is to test some previously prepared mixed matrix membranes for their ability to separate hydrogen mixtures. In this study, MMMs were prepared via a combination of a poly(o-hydroxyamide) (HPA) and a microporous polymer network and tested as hydrogen purification materials. First, the hydrogen permselectivity versus CH_4_, CO_2_, and N_2_ was evaluated in these novel MMMs. Subsequently, these MMMs were thermally treated to convert the HPA matrix to a polybenzoxazole one (β-TR-PBO), thus generating the corresponding β-TR-PBO MMM (TR-MMMs).

## 2. Materials and Methods

The 6FCl-APAF poly(*o*-hydroxyamide), HPA, was synthesized in our lab following a methodology previously described [35]. To prepare the mixed matrix membranes (MMMs), a porous polymer network (PPN) was synthetized prior to this work by reacting triptycene (TRP) and 2,2,2-trifluoroacetophenone (TFAP), according to the methodology described by Lopez-Iglesias et al. (2018) [35,38]. Porous features of this PPN were also described in the article abovementioned. In particular, a moderately high BET area of 655 m^2^/g was measured by Lopez-Iglesias et al. [38] by N_2_ adsorption isotherms.

MMMs were prepared by a solvent evaporation process. Thus, 500 mg of HPA was dissolved in 5 mL of NMP (10%) and filtered through a 3.1-µm fiberglass filter (Symta, Madrid, Spain) to remove dust particles. Concomitantly, an amount of PPN particles was dispersed in 0.5 mL of *N*-methyl-2-pyrrolidone (NMP) (10% *w*/*w* suspension of PPN). The suspension particles were stirred for 2 h and then sonicated for 20 min (40 cycles of 20 s of sonication followed by 10 s without ultrasound, using 30% amplitude) using a 130-W ultrasonic probe Branson 450 Digital Sonifier (Marshall Scientific, Hampton, NH, USA) to promote the breaking of the PPN-agglomerated particles and to obtain a homogeneous dispersion of the particles. To achieve high polymer–particle compatibility and thus promote higher affinity in order to improve the transport properties of MMMs, the priming technique was employed [40], which involves adding a small amount of polymer to the filler suspension before incorporating the particles into the polymer solution. Thus, the PPN particles were primed by adding approximately 10% of the total volume of the polymer solution and then sonicated at 30% amplitude for 5 min [39]. Finally, the polymer matrix solution and PPN dispersion were combined and stirred again for 1 h to obtain a homogeneous distribution of the PPN particles within the polymer matrix solution.

The polymer solutions were cast onto well-leveled glass plates and were kept at 60 °C overnight on the plate at atmospheric pressure, followed by the next thermal treatment steps under vacuum: 2 h at 80 °C, 1 h at 100 °C, 2 h at 120 °C, and 12 h at 180 °C. Finally, the membrane samples were slowly cooled down to room temperature in the vacuum oven.

**Figure 1:**
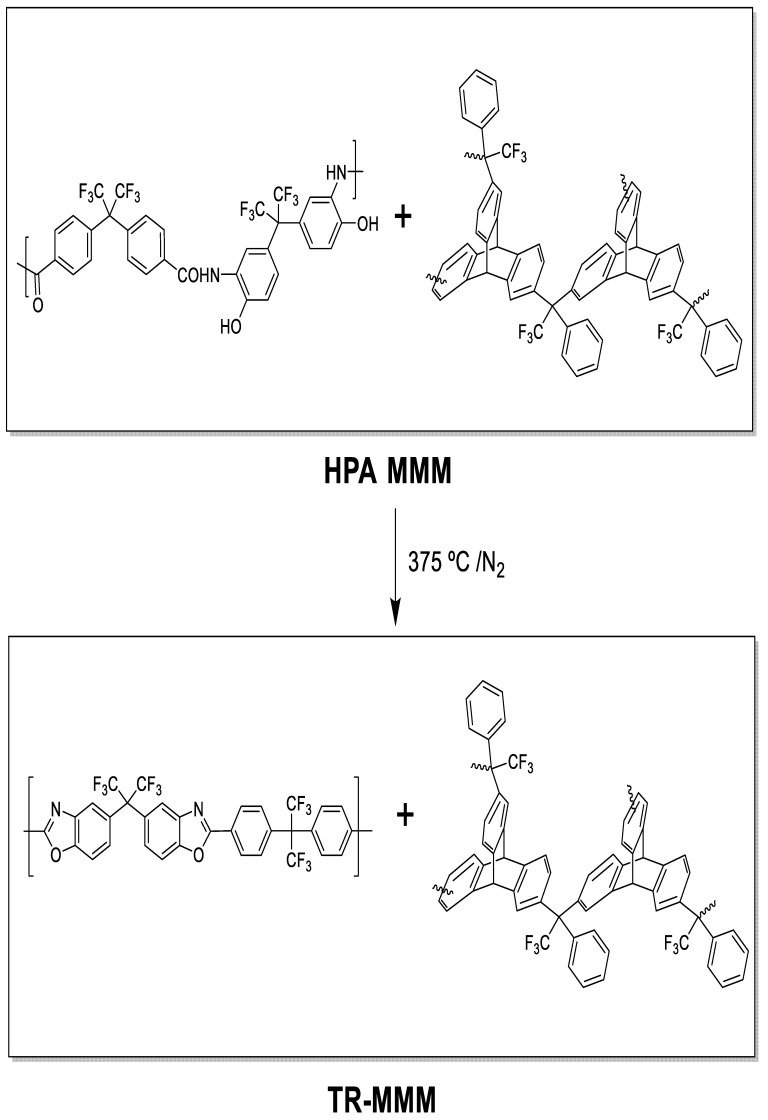
HPA-MMMs and their corresponding thermally rearranged TR-MMMs.

To obtain β-TR-PBO mixed matrix membranes (TR-MMMs), the HPA-PPN MMM membranes were thermally treated through a solid-state rearrangement reaction [35,38] by initially heating up to 250 °C at a rate of 5 °C/min, maintaining this temperature for 15 min, and further heating to 375 °C at a rate of 5 °C/min, maintaining this temperature for 15 min. This method was previously optimized to obtain complete thermal rearrangement [31] of the polymeric matrix and similarly at higher temperatures to obtain α-TR-PBO from ortho-hydroxypolyimides [41]; it was shown that PPN does not degrade by extensive TGA analyses. The procedure was carried out under a N_2_ atmosphere in a Carbolite CTF 12/65/700 single-zone pyrolizer furnace (Carbolite-Gero, Hope, UK) equipped with a quartz tube and using ultra-high-purity nitrogen flow rate at 900 mL/min. The 6FCl-APAF (HPA-PPN) MMMs and its corresponding TR-MMMs are depicted in Figure 1.

## 3. Membrane Morphology

### 3.1. Methodology

Scanning electron microscopy (SEM) images were taken with a QUANTA 200 FEG ESEM (Thermo Fisher Scientific, Waltham, MA, USA) on Au-metallized samples operating at an acceleration voltage of 1.5 kV in high vacuum and using the detection of secondary electrons method. The samples were cryogenically fractured using liquid N_2_ to obtain images of the cross-section.

Atomic Force Microscopy (AFM) surface scans were obtained using a Nanoscope Multimode IIIa^®^ from Digital Instruments (Veeco Metrology Inc., Santa Barbara, CA, USA) in tapping mode. Roughness and Power Spectral Density (PSD) [42] were analyzed using NanoScope Software Version 5.30 (Veeco Metrology Inc., Santa Barbara, CA, USA). The so-called E scanner (horizontal and vertical ranges of 10 and 2.5 μm, respectively) was used here. The scanned areas went from 500 × 500 nm to 10 × 10 μm.

Before AFM imaging, the samples were previously cleaned by using low-power radiofrequency plasma. An expender Plasma Cleaner with a Plasma Flow Gas Mixer and a Digital Thermocouple Gauge Control Unit (HARRICK PLASMA, Ithaca, NY, USA) were used for this purpose. Samples were placed on a quartz plate in an Ar atmosphere under a flow of 0.5 L/min at 550 mTorr at a power of 10.2 W during 10 min.

### 3.2. Results

**Figure 2:**
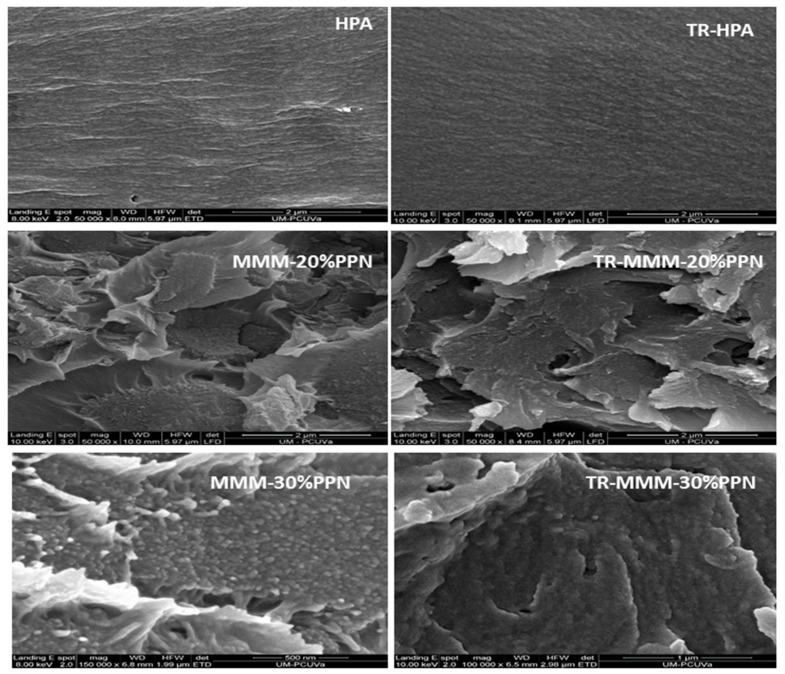
Comparison of cross-section tensile fracture surface of MMMs by SEM images before and after thermal treatment.

A good compatibility between the continuous phase and the dispersed phase is seen in Figure 2, which shows some examples of SEM pictures for the studied membranes. They seem to have a good enough polymer–filler compatibility without significant agglomeration [43]. The cracks appearing in all images of MMMs are due to the cryogenic fracturing of the membrane cross-sections [39].

HPA and thermally rearranged HPA and TR-HPA were compared with the corresponding MMMs to show the influence of the load on morphology. In fact, these very slight differences could be due to changes in the packing of the polymeric matrix induced by the thermal process of the filler that could modify the matrix–filler interfaces. Some effect of the presence of the filler on the evaporation of possible residual solvents cannot be discarded [36].

Agglomeration and detaching of the filler were observed at 40% filler, which led to a clear decrease of permeability (not observed at 30% PPN load, as will be shown below). This fact could be attributed to a stiffening of the linear macromolecular chains, leading to an increase in tension around the particles [35] and reducing the filler-to-matrix compatibility overruled by filler-to-filler affinity.

MMMs form a three-phase system: (i) the filler phase, (ii) the polymer phase, and (iii) the rigidified (or less rigid) formed interface due to the nature of the polymer–filler interaction and the stress generated during membrane fabrication [44]. In this context, as mentioned, solvent evaporation is an important factor to be considered. In effect, according to Mahajan et al. [45], membrane size shrinks during the solvent evaporation process. This shrinkage could generate high stress in the material and cause the filler to detach. This could result in the increase of membrane defects, leading to the formation of heterogenous voids at the interfaces. However, in some cases, the polymer chains can conform to the filler surface and relieve the stress, thus obtaining a defect-free interface [46]. In the case of TR-MMMs, in addition to combining the properties of the disperse and continuous phases, the membranes present the properties conferred by the thermal treatment, which form microcavities [28] that mainly favor the diffusion of light gases when compared with HPAs. Moreover, when a good enough contact between the continuous and dispersed phases is achieved, a still fair selectivity would be obtained for the corresponding TR-MMMs. This could happen when there are low residual stress forces that relax when the membranes are exposed to temperatures above the T_g_ of the polymer matrix [36].

**Figure 3:**
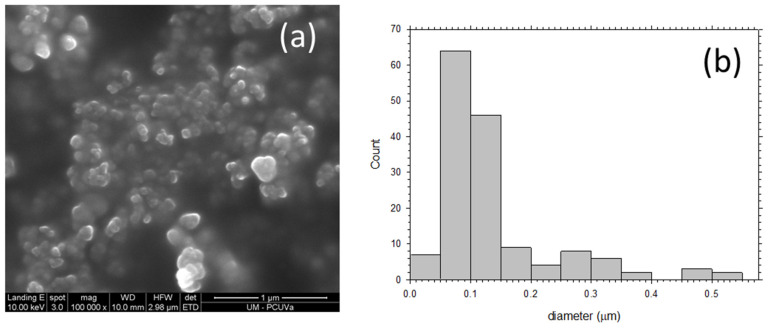
(**a**) SEM picture of deposited PPN and (**b**) grain size distribution.

To obtain SEM micrographs of the PPN particles used as filler, the following procedure was carried out: PPN particles (30 mg) were added to 1 mL of ethanol and stirred for 2 h followed by sonication for 20 min (40 cycles of 20 s of sonication followed by 10 s without ultrasounds, using 30% amplitude). The suspension was then stirred for 48 h and again sonicated for 20 min using the same conditions commented above. Finally, suspensions were prepared by diluting (dilution factor of 10) twice. Images such as those seen in Figure 3 were obtained. It can be seen that the most probable size of the mostly globular PPN particles is 75 nm but some particles are as large as 500 nm in diameter.

**Figure 4:**
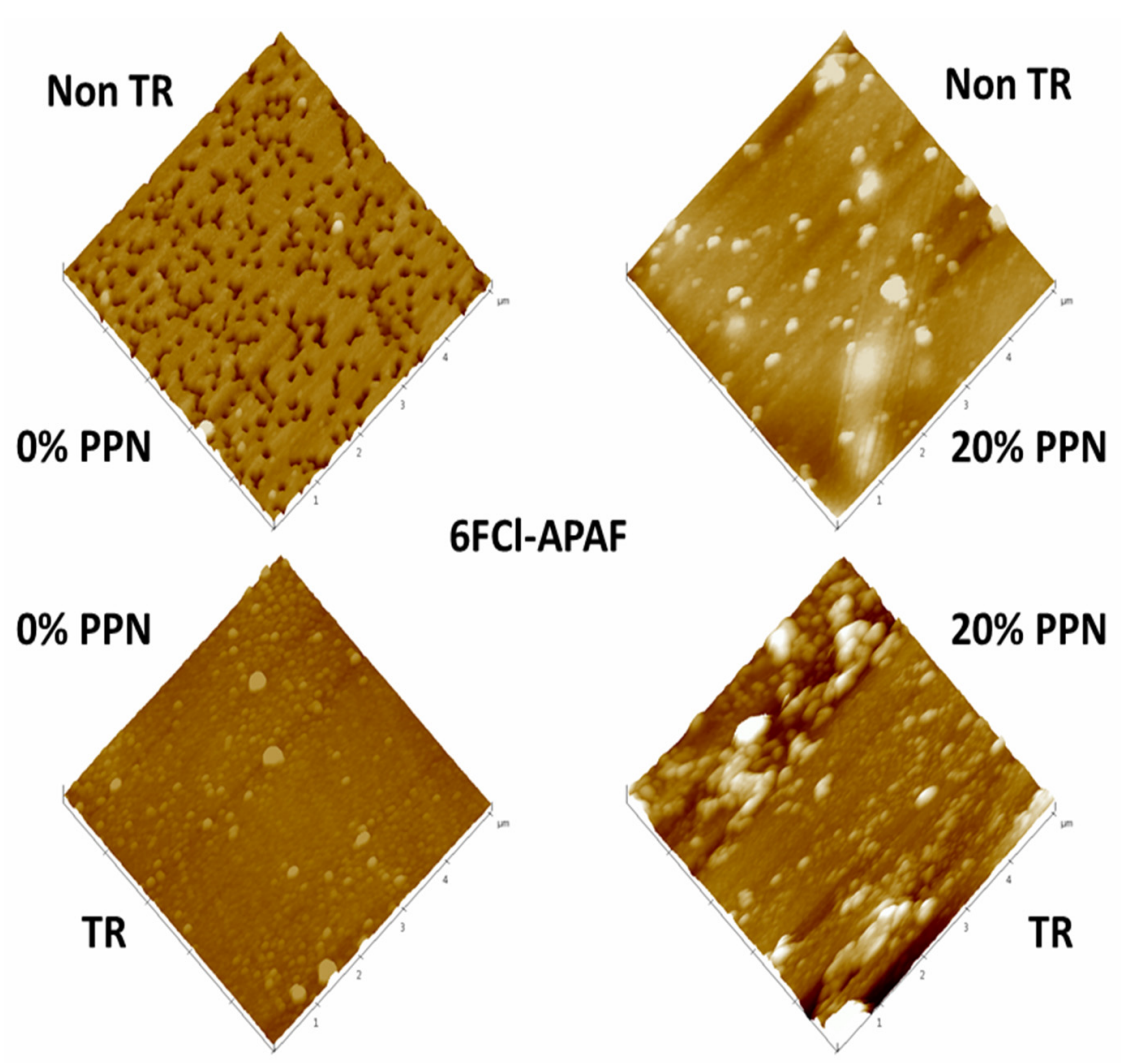
AFM pictures of pure HPA and HPA-PPN MMM.

Examples of 3D AFM images are shown in Figure 4 for samples previously cleaned by using low-power radiofrequency plasma as mentioned before. The corresponding fractal dimensions and roughness [42] are shown in Table 1. Fractal dimension is always very close to 2, which would indicate a fairly planar surface; although, slightly better planarity (fractal dimension closer to 2) can be noted when PPN content increases and when thermal rearrangement is performed. Concerning roughness, it decreases before thermal rearrangement when increasing the PPN content and increases after thermal rearrangement when PPN content increases. This different behavior could be due to an increasing rigidity of the polymeric matrix chains after thermal rearrangement that hinders the appropriate adaptation of the matrix to accommodate PPN [35].

## 4. Gas Transport Properties

### 4.1. Methodology

Single gas (except H_2_) permeability coefficient measurements for HPA, MMMs, and TR-MMMs were determined using a constant volume apparatus at 35 °C and an upstream pressure of 3 bar, as described elsewhere [35]. H_2_ permeability was measured at The University of Texas at Austin facilities using also a constant volume apparatus described by Lin and Freeman at the same pressure and temperature [47]. CO_2_ was also measured by this way with completely coincident results, within the error ranges, thus, assuring coherence between both methods of measurement. The constant volume method has been described and studied in the literature, for example, quite comprehensively by Flaconnèche et al. [48].

To rule out the presence of pinholes in the membrane, helium permeability was measured at three different pressures (1, 2, and 3 bar) before placing the membrane in the permeation cell in the constant-volume devices. Membranes without pinholes were kept overnight under vacuum before the determination of their permeability for He, H_2_, N_2_, O_2_, CH_4_, and CO_2_. The permeability coefficient, *P*, in Barrer, was obtained using the following expression:

(1) $$P=\frac{273}{76}\frac{V\ell }{ATp_{0}}\left(\frac{dp}{dt}\right)$$

$$1\;\mathrm{Barrer}=10^{-10}\left[{\mathrm{cm}}^{3}\left(\mathrm{STP}\right)\cdot \mathrm{cm}/{\mathrm{cm}}^{2}\cdot s\;\cdot \mathrm{cm}\;\mathrm{Hg}\right]=3.35\times 10^{-16}\left[\mathrm{mol}\cdot m/m^{2}\cdot s\cdot \mathrm{Pa}\right]$$

where *V* is the downstream volume in cm^3^, ℓ is the thickness of the membrane in cm, *A* the effective area of the cell in cm^2^, *T* is the temperature in K, po is the upstream pressure in cmHg, and *dp*(*t*)/*dt* is the variation in the downstream pressure with time. The thickness of the membrane was measured before measurement of the permeability coefficient into the cell. The ideal selectivity for a gas pair was calculated from the ratio of their pure gas permeabilities, *P_A_* and *P_B_*, as follows:

(2) $$\alpha_{A/B}=\frac{P_{A}}{P_{B}}$$

where *P_A_* and *P_B_* are the permeability coefficients of the pure gases, *A* and *B*, respectively.

In accordance with time-lag theory [49,50,51], the diffusion coefficient is inversely proportional to time lag as

(3) $$D=\frac{\ell^{2}}{6t_{\ell }}$$

Here, $t_{\ell }$ is time lag (time for zero pressure extrapolated from the linear pressure versus time plot). As P=DS, this allows the evaluation of solubility, *S*, as well.

### 4.2. Results

Permeability values (*P*) of H_2_, N_2_, CH_4_, and CO_2_ at 3 bar and 35 °C were measured for the polymeric matrix alone, precursor MMMs, and their corresponding β-TR-PBO MMMs (TR-MMMs) along with their ideal selectivity (α) for some selected gas pairs.

**Figure 5:**
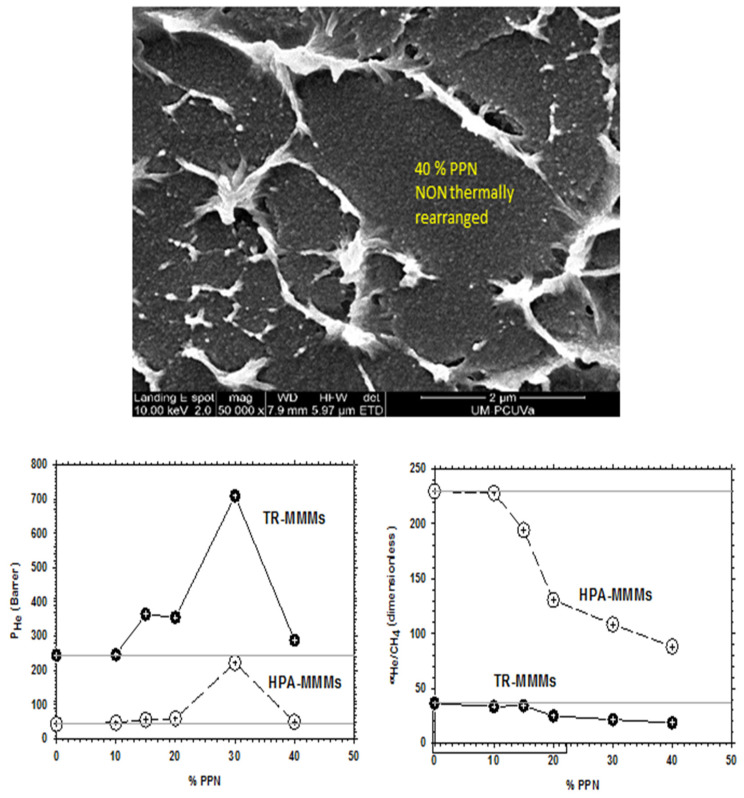
SEM picture of pure HPA along with He permeability and He/CH_4_ selectivity for the HPA-PPN MMM and their TR counterparts as a function of PPN content.

As already mentioned in Section 2, for PPN contents over 30%, permeability decreases with a constant trend to decrease selectivity slightly and, simultaneously, bigger aggregates of the filler appear. These outcomes, as an example, are shown in Figure 5 for the He/CH_4_ pair of gases. This behavior discourages the use of such relatively high PPN contents.

**Figure 6:**
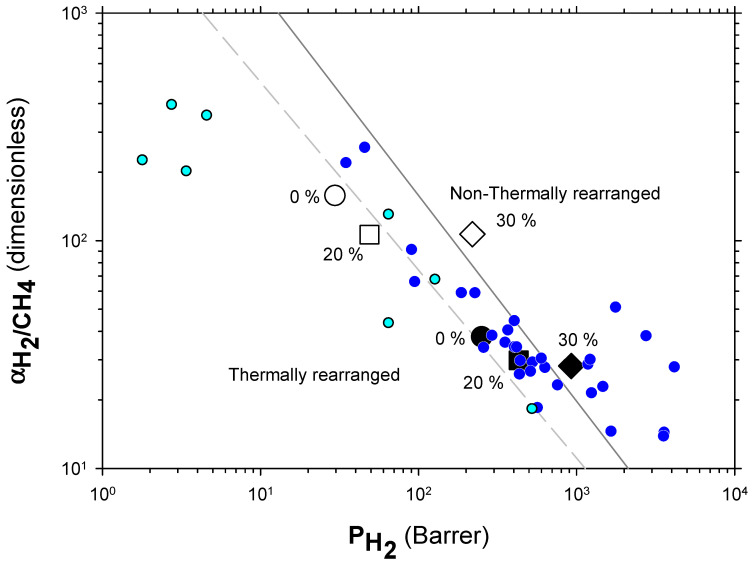
Robeson plot for H_2_/CH_4_. Non-TR (HPA) membranes are shown as open symbols, while TR (TR-PBO) membranes are shown as closed symbols. Circles (●), squares (■), and diamonds (◆) correspond to 0%, 20%, and 30% PPN loading, respectively. The dashed and continuous lines represent the 1991 and 2008 upper bounds, respectively. Dark-blue symbols correspond to α-TR polymers and light-blue ones to some β-TR polymers [54].

**Figure 7:**
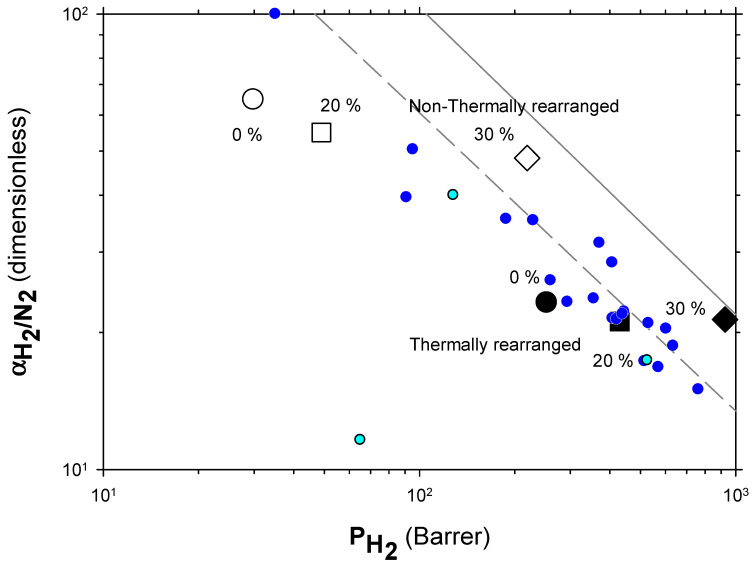
Robeson plot for H_2_/N_2_. Non-TR (HPA) membranes are shown as open symbols, while TR (TR-PBO) membranes are shown as closed symbols. Circles (●), squares (■), and diamonds (◆) correspond to 0%, 20%, and 30% PPN loading, respectively. The dashed and continuous lines represent the 1991 and 2008 upper bounds, respectively. Dark-blue symbols correspond to α-TR polymers and light-blue ones to some β-TR polymers [54].

**Figure 8:**
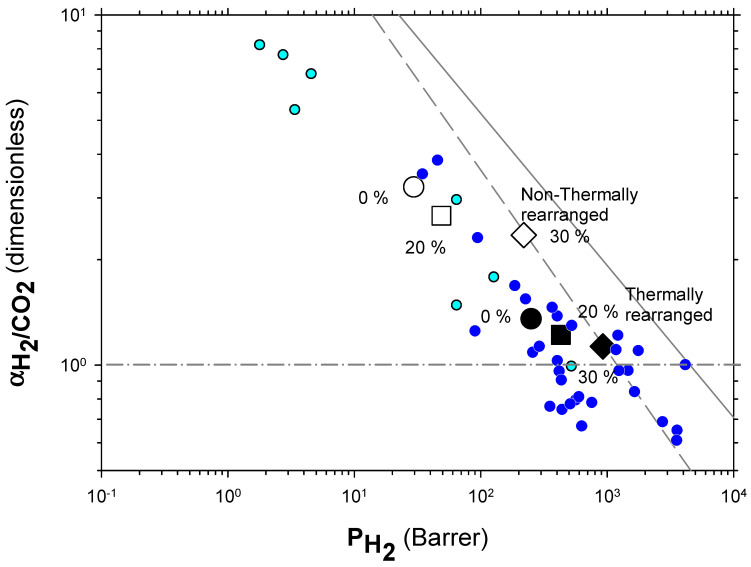
Robeson plot for H_2_/CO_2_. Non-TR (HPA) membranes are shown as open symbols, while TR (TR-PBO) membranes are shown as closed symbols. Circles (●), squares (■), and diamonds (◆) correspond to 0%, 20%, and 30% PPN loading, respectively. The dashed and continuous lines represent the 1991 and 2008 upper bounds, respectively. Dark-blue symbols correspond to α-TR polymers and light-blue ones to some β-TR polymers [54].

The Robeson plots [52,53] (double log plot of ideal selectivity versus permeability of the most permeable gas in a given pair) for the H_2_/CH_4_, H_2_/N_2_, and H_2_/CO_2_ are shown in Figure 6, Figure 7 and Figure 8. Note that the Robeson permeability–selectivity trade-off lines, i.e., the upper bounds, are shown as straights lines (dashed lines for the 1991 upper bound [52] and continuous lines for the 2008 upper bound [53]). Data for other several thermally rearranged (TR) polymers as collected by Kim and Lee [54] (not including copolymers nor MMMs) are also shown in these plots for the sake of comparison.

The first conclusions that can be extracted from these plots is that high-PPN content leads to very high permeability with a slight decrease in selectivity. However, it should be noted that a good compromise between these two parameters, especially for the H_2_/CH_4_ separation, is seen. Good permeability and selectivity properties for the H_2_/N_2_ pair are also evident. The corresponding points in the Robeson’s plot exceed the 2008 Robeson upper bound for high-PPN loads and the pair H_2_/CH_4_. For the H_2_/N_2_ pair, the 2008 Robeson’s bound is approached. The thermal rearrangement of the mixed matrix membranes increased permeability, but simultaneously decreased their selectivity substantially. In all cases, the permeability versus selectivity compromise is fairly good for the membranes before their thermal rearrangement as well. It is important to note that for all the gas pairs shown in Figure 6, Figure 7 and Figure 8, our β-TR polymers stand out from those more conventional ones shown by Kim and Lee [54]. Actually, they show permeabilities and selectivities similar to those typically given by α-TR polymers. In fact, the representative clouds of both kinds of thermally rearranged polymers partially intermingle.

**Figure 9:**
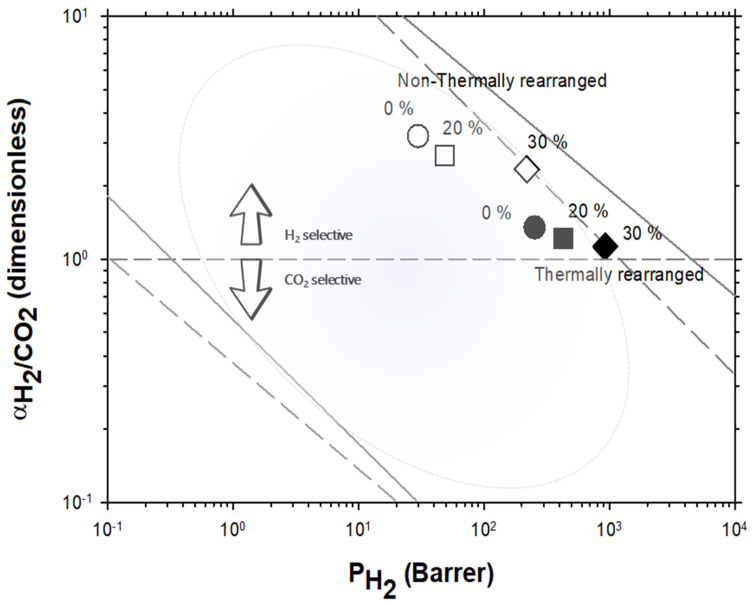
Robeson plot for H_2_/CO_2_ compared with some commercial polymeric membranes [55] (stars). Acronyms: cellulose acetate, CA; polysulfone, PSF; polyethersulfone, PES; polyimide, PI; polyetherimide, PEI; Polydimethylsiloxane, PDMS: polymethylpentene, PMP; poly(phenylene oxide), PPO; polystyrene, PS; ethyl cellulose, EC. Non-TR membranes are shown as open symbols, while TR membranes are shown as closed symbols. Circles (●), squares (■), and diamonds (◆) correspond to 0%, 20%, and 30% PPN loading, respectively. The dashed and continuous lines represent the 1991 and 2008 upper bound, respectively [33,52,53].

For the H_2_/CO_2_ gas pair, the decrease in selectivity induced by thermal rearrangement led to almost vanishing selectivity (close to 1); although, before thermal rearrangement, the permeability versus selectivity compromise was objectively better. In Figure 9, the Robeson’s plot for the H_2_/CO_2_ pair is shown along with some examples of existing polymeric membranes, as shown by Huang et al. [55].

**Figure 10:**
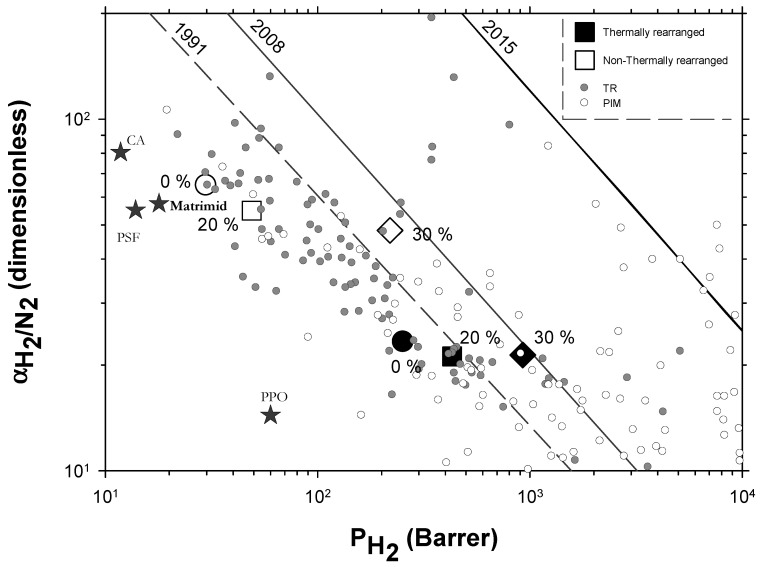
Robeson plot for H_2_/N_2_ including some commercial polymeric membranes [56] (stars). Acronyms: cellulose acetate, CA; polysulfone, PSF; poly(phenylene oxide), PPO; Matrimid. Other TR membranes correspond to solid gray circles while some PIM membranes correspond to open circles [30]. Our membranes are shown as open symbols without TR, while TR membranes are shown as closed symbols. Circles (●), squares (■), and diamonds (◆) correspond to 0%, 20%, and 30% PPN loading, respectively. Lines represent the 1991, 2008, and 2015 upper bounds.

Very few extensive analyses of TR membranes for H_2_ or H_2_ mixtures can be found in the literature other than in the works of Dong and Lee [30] and Kim and Lee [54]. Nevertheless, a recent, quite exhaustive review was published by Bandehali et al., which included Polymers of Intrinsic Microporosity (PIM) and thermally rearranged (TR) polymers (including MMMs made by using them as polymeric matrices with different fillers and copolymers) [30] with information on hydrogen separation when mixed with nitrogen only. In order to place our membranes in this more general context, we include their data in Figure 10 where some commercial membranes [56] and the so-called 2015 upper bound [57] are also shown. It should be pointed out that different copolymers and MMMs and different ageing times and treated PIMs and TRs are shown by Bandehali et al. [30] and they do not distinguish between α-TR and β-TR. It is generally accepted that PIMs and TR polymers have outstanding gas transport properties, as to delineate the state-of-the-art in polymeric membranes. PIMs tend to have the highest permeability while TR polymers show higher selectivities for differently sized gases, which is the case for H_2_/N_2_. Although, it is worth noting that most of the TR membranes are well below the 2008 upper bound. Our membranes go from relatively high selectivity and low permeability before thermal rearrangement to the zone of relatively lower selectivity and high permeability after thermal rearrangement. Within both these zones, permeability improves when the filler content increases with only slightly decreasing selectivity.

**Figure 11:**
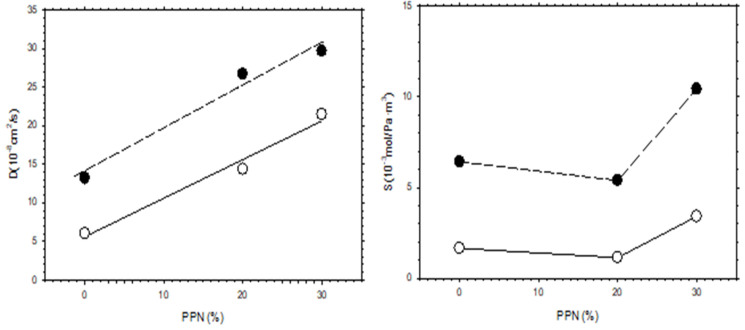
H_2_ diffusivity and solubility as a function of PPN content. Filled symbols correspond to thermally rearranged MMMs while the open symbols correspond to non-TR-MMMs.

**Figure 12:**
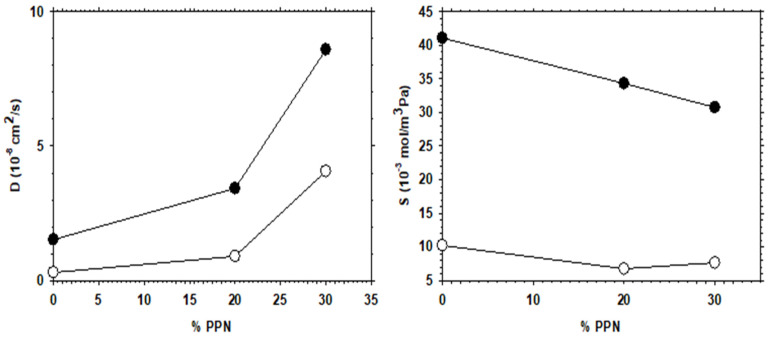
CO_2_ diffusivity and solubility as a function of PPN content. Filled symbols correspond to thermally rearranged MMMs.

The increase in permeability of H_2_, as a function of PPN loading and/or after thermal rearrangement is evident, as seen in Figure 11, where H_2_ diffusivity and solubility are shown. It appears clear that permeability $\left(P_{A}=D_{A}S_{A}\right)$ increases mainly due to the contribution of diffusivity, while the gas solubility is slightly more relevant after thermal rearrangement. For comparison purposes, the corresponding CO_2_ diffusivity and solubility are shown in Figure 12.

Comparing the diffusivity and solubility trends of CO_2_ and H_2_, it was observed that the diffusivity values of H_2_ and CO_2_ increased both by increasing PPN content and by the thermal rearrangement process. In turn, increasing PPN loadings led to a constant value, or slightly higher solubility for H_2_, while the solubility of CO_2_ decreased slightly. Since these trends lead to differences in CO_2_ and H_2_ permeability and, thus, to changes in selectivity, it can be concluded that diffusivity, rather than solubility, should control the separation process. This can be interpreted in terms of the formation of more transport pathways when more PPN is added, probably through the interfaces surrounding the PPN domains.

The increase in permeability of H_2_ along with the small decline of selectivity (see Figure 6, Figure 7 and Figure 8) could indicate an increase in the non-transport-restrictive, fractional-free volume of the materials. In a previous work [35], it was shown that PPN addition decreased intersegmental distances and increased rigidity. These increments were mainly due to increasing the internal volume of the voids, and the opening and widening of the void-to-void gap channels. Moreover, the tendencies observed in the Robeson’s plots (Figure 6, Figure 7 and Figure 8), according to the classification made by Moore and Koros [58], could correspond to the presence of voids within certain loose shells established in the polymer–filler interfaces surrounding the filler. In this respect, the 40% PPN case shown in Figure 5 should correspond to the inaccessibility of most of this additional free volume due to the formation of bigger aggregates. Therefore, the changes in permeability can be attributed mostly to changes in the polymer–filler interactions.

## 5. Conclusions

Mixed matrix membranes (MMMs) were obtained by combining different amounts of a microporous polymer network (PPN) with an o-hydroxypolyamide (HPA) matrix of 6FCl-APAF. The effect of the thermal treatment on HPA and the HPA-MMMs, with different PPN loadings, was studied. It was seen that the resulting MMMs suffered complete thermal rearrangement at 375 °C to give the corresponding polybenzoxazole MMMs (TR-MMMs) observing no degradation of the PPN load.

PPN contents up to 30% increased permeability with only a limited level of aggregation, as confirmed by SEM and AFM imaging that confirmed the presence of smooth surfaces with limited roughness.

The MMMs showed very high H_2_ permeabilities with excellent H_2_ selectivities for the H_2_/CH_4_ and H_2_/N_2_ gas pairs. The values for the H_2_/CO_2_ mixture without any thermal treatment were worse than for the other gas pairs, but superior to those seen for other common polymer membranes. After the thermal rearrangement, selectivity was almost totally lost for the H_2_/CO_2_ pair.

In all cases, thermal rearrangement augments permeability and decreases selectivity. An increase in PPN loading showed similar effects, as it increased permeability and slightly decreased selectivity. These effects appear to originate from the formation of diffusive paths across the matrix–filler interfaces.

It is worth noting that for the gas pair analyzed here, the non-thermally-rearranged matrices, HPA-MMMs, provide a good selectivity versus permeability compromise in the zones of high selectivity and close to, or even over, the Robeson’s bounds. Thus, when dealing with a specific application, we can use the non-thermally-treated membranes to limit the number of steps needed for a given recovery, or we can choose to apply the thermal rearrangement process to limit the membrane area required for a given productivity.

## Figures and Tables

**Table 1:** Fractal dimensions, d_fr_, and roughness, R_q_, for the MMMs studied both before and after thermal rearrangement and with different PPN loads.

		Membranes	
	PPN Content (%)	Before TR	After TR
d_fr_	0	1.76	1.78
	20	1.62	1.93
	30	1.92	2.03
R_q_ (nm)	0	14.90	4.32
	20	4.77	5.06
	30	3.45	12.70

## Data Availability

The raw and processed, data, and procedures required to reproduce these findings have been fully described in this paper and/or referenced. Additional data cannot be shared at this time due to technical limitations and are not needed to reproduce our results.
